# A different perspective: using interactive virtual reality (IVR) for psychiatry training

**DOI:** 10.1192/bjo.2021.115

**Published:** 2021-06-18

**Authors:** Huw Evans, Sophie Young, Josh Whitehurst, Abdul Madadi, Joanne Barton

**Affiliations:** 1 Midlands Partnership NHS Foundation Trust; 2 Shrewsbury and Telford NHS Trust; 3 North Staffordshire Combined Healthcare NHS Trust

## Abstract

**Aims:**

To evaluate the potential of interactive virtual reality in teaching and training Postgraduate Psychiatry Trainees in the Keele Cluster

**Background:**

Face to face supervised clinical experience will always be the best way to train and learn, followed by using simulated patients in practice scenarios allowing a safe environment in which to practice and train without risk. However, the practicalities of a busy NHS often mean that the expense and time required for both of these are not possible and often PowerPoints and handouts in induction are used to prepare new starters in Psychiatry, which is clearly suboptimal. Interactive Virtual Reality (IVR) allows trainees to not only be immersed in a simulation but take control, choosing the direction of questioning for example. It also allows the training to be easily repeated and scaled to any number of students, anytime and anywhere there is an internet connection.

**Method:**

Following successful funding from the RCPsych General Adult Faculty we chose three common scenarios that a new started in Psychiatry would face. These included acute agitation/rapid tranquilisation, a patient wishing to leave/section 5(2) and a patient with tachycardia following clozapine initiation. Using established guidelines and literature, in conjunction with feedback from subject matter experts and practicing clinicians, scenarios were written. We then researched the best hardware and software to make this possible, ensuring that the resources required were realistic to allow accessibility to as many trainees as possible.

**Result:**

Creating IVR is challenging but an engaging medium. Achieving consensus on the training material is time consuming yet paramount to a good training session. Producing high quality videos is extremely resource intensive requiring large amounts of computing power and storage. However, the outcome is an engaging and practical alternative to face to face training.

**Conclusion:**

The possibilities for IVR for are vast. For example, trainees can practice different methods of asking questions (e.g. open vs closed) and how this affects the outcome. Training could be produced centrally and then shared, allowing best practice to be disseminated. It could improve and standardise induction, especially considering the expanding workforce. It could also improve recruitment, allowing an immersive experience of Psychiatry to those who would otherwise be unable to obtain shadowing. It also has a role in patient safety – demonstrating common scenarios that the trainee may face allowing them to practice in a safe environment.

